# Establishment of Mouse Models of Psoriasis with Blood Stasis Syndrome Complicated with Glucose and Lipid Metabolism Disorders

**DOI:** 10.1155/2019/6419509

**Published:** 2019-11-12

**Authors:** Ying Luo, Yi Ru, Huaibo Zhao, Liu Liu, Seokgyeong Hong, Xiaoying Sun, Le Kuai, Yi Lu, Meng Xing, Xi Chen, Jiankun Song, Yue Luo, Xiaoya Fei, Yaqiong Zhou, Hongjin Li, Bin Li, Xin Li

**Affiliations:** ^1^Department of Dermatology, Yueyang Hospital of Integrated Traditional Chinese and Western Medicine, Shanghai University of Traditional Chinese Medicine, Shanghai 200437, China; ^2^Institute of Dermatology, Shanghai Academy of Traditional Chinese Medicine, Shanghai 201203, China

## Abstract

**Background:**

Psoriasis has been reported as a high-risk factor for quality of life and survival rate in patients with metabolic disorder. However, there is no animal model for studying this disease. This study aimed to establish and evaluate mouse models of psoriasis with blood stasis syndrome (which is a key to psoriasis pathogenesis, according to Chinese Medicine) complicated with metabolic disorders.

**Method:**

Forty-five C57BL/6 mice were randomly divided into the blank control (Control), psoriasis (Imiquimod (IMQ)), psoriasis with metabolic disorders (IMQ + streptozotocin (STZ)), psoriasis with blood stasis syndrome (BSS) (IMQ + BSS), and psoriasis with blood stasis syndrome complicated with metabolic disorders (IMQ + STZ + BSS) groups (*n* = 9 mice/group). Psoriasis lesions were induced using IMQ cream (on both the ears and back, except in the Control group). Mice of the IMQ + BSS group were fed a half-fat, high-sugar diet and stimulated with ice-water swimming every day. Mice of the IMQ + STZ group were fed a half-fat, high-sugar diet and injected with STZ. Mice of the IMQ + STZ + BSS group were subjected to the same treatments as the IMQ + STZ and IMQ + BSS groups. After induction, the mice in each group were observed for vital signs, ear thickness, body weight, and psoriasis area and severity index (PASI) score. The mice were fasted for 12 h before determination of related laboratory serum indexes. Dorsal skin lesions, aortic arch pathology sections, and signal transducer and activator of transcription 3 (STAT3) were examined by H&E staining and immunohistochemistry.

**Results:**

Laboratory indexes in the four model groups were significantly different from those in the Control group (*p* < 0.01); indicators of the IMQ + STZ, IMQ + BSS, and IMQ + STZ + BSS groups showed varying degrees of difference from those of the IMQ group.

**Conclusions:**

The established mouse models of psoriasis blood stasis syndrome complicated with glucose and lipid metabolism disorders met the clinical and Chinese Medicine characteristics, and thus they could be used as animal models in future studies of psoriasis complicated with glucose and lipid metabolism disorders.

## 1. Introduction

Psoriasis is a clinically common chronic relapsing inflammatory skin disease, and it is called “Baibi” or “Songpixian” in Chinese Medicine (CM). A previous study [1] has shown that patients with psoriasis have obvious local microcirculation disorders and varying degrees of vascular endothelial injury, which cause increased blood viscosity and microvascular disorders [2]. In addition, significantly abnormal lipid metabolism is shown by patients with psoriasis [3]. Elevated cholesterol, triglyceride, and low-density lipoprotein (LDL) levels can accelerate the adhesion and aggregation of platelets as factors affecting psoriasis [4]. Augustin et al. [5] found in a retrospective study that the incidence of metabolic syndrome in patients with psoriasis is much higher than that in healthy people, and metabolic syndrome is a high risk factor for cardiovascular and cerebrovascular diseases. Currently, the available treatment methods for psoriasis are mainly based on biological and systematic therapies, supplemented by physical therapy. However, some adverse reactions are associated with the above therapies, such as abnormal glucose and lipid metabolism, which undoubtedly worsen the condition of patients with psoriasis complicated with metabolic disorders [6, 7]. Therefore, studies on models of psoriasis complicated with metabolic disorders have important significance in the development of drugs for the treatment of psoriasis, which may reduce the incidence of complications and improve the quality of life and survival rate of patients.

In CM, blood is believed to be the disease location of psoriasis, given that the classic CM syndromes of psoriasis include “blood heat,” “blood stasis,” and “blood deficiency” [8]. Moreover, phlegm, heat, stasis, and asthenia represent the key pathomechanism of metabolic disorders [9]. In the early stage of psoriasis, blood heat is abundant and causes obstruction of the muscle surface. In the later stage, blood heat is concentrated in the blood, and blood circulation is not smooth and turns into static blood. Alternatively, the chronic disease consumes qi and blood, and the deficiency of qi and blood can cause blood stasis, all of which can lead to the occurrence of metabolic syndrome, in which blood stasis is common [10]. Blood stasis syndrome occurs throughout the onset of psoriasis [11]. When patients with psoriasis are in the state of blood stasis syndrome for a long time, static blood is latent in the meridians, viscera, and limbs, forming various symptoms similar to glycolipid metabolism disorders, such as diabetes and metabolic syndrome [12]. At present, there are neither reports nor studies on animal models of psoriasis with blood stasis syndrome complicated with metabolic disorder, thereby hindering the development of new clinical drugs for this disease. Therefore, this study aimed to establish mouse models presenting the characteristics of psoriasis with blood stasis and glycolipid metabolism disorders. IMQ, STZ, and ice-water swimming were used to induce psoriasis with blood stasis syndrome complicated with glycolipid metabolism disorder in mice.

## 2. Materials and Methods

### 2.1. Material and Apparatus

#### 2.1.1. Animals

Forty-five male C57BL/6 mice weighing 25 ± 3 g were provided by Shanghai Medical Experimental Animal Center (SCXK Shanghai 2013–0016, Shanghai, China). The animals were maintained under a standard temperature of 23 ± 2°C and 12-h light-dark cycle. The mice were grouped into three mice per cage with free access to standard diet and water. All procedures were reviewed and approved by the Scientific Research Department of Yueyang Hospital affiliated to Shanghai University of Traditional Chinese Medicine (animal certificate nos. 2015000544758, 2015000546963, and 2015000549944). All procedures were reviewed and approved by the Ethics Committee of Yueyang Hospital affiliated to Shanghai University of Traditional Chinese Medicine (no. 17772).

#### 2.1.2. Groups

All mice were randomly divided into five groups: blank control group (Control group), psoriasis group (IMQ group), psoriasis with metabolic disorders group (IMQ + STZ group), psoriasis with blood stasis syndrome (BSS) group (IMQ + BSS group), and psoriasis with blood stasis syndrome complicated with metabolic disorders group (IMQ + STZ + BSS group), with nine mice in each group.

#### 2.1.3. Experimental Apparatus

Low-density lipoprotein-cholesterol (LDL-C) and total cholesterol testing kits were purchased from Nanjing Jiancheng Institute of Biological Engineering (Jiangsu, China). Vascular endothelial growth factor (VEGF), endothelin-1 (ET-1), insulin, and mouse C-peptide testing kits were obtained from Shanghai Xinyu Biotechnology Co., Ltd. (Shanghai, China), and the rapid glucose meter was purchased from Bayer (Baiankang 1816, Leverkusen, Germany).

#### 2.1.4. Drugs

IMQ cream was purchased from Sichuan Mingxin Pharmaceutical Co., Ltd. (Sichuan, China; batch number: National Drug Approval Letter H20030128). Norepinephrine (NE) adjusted to 0.1 g/l was obtained from Shanghai Wellhope Pharmaceutical Co., Ltd. (Shanghai, China; batch number: National Drug Approval Letter H31021177). STZ powder (batch number: Cas18883-66-4) was purchased from Beijing Boai Port Trade Center (Beijing, China). STZ buffer was configured from liquid A (0.1 M citric acid solution, 1.05 g constant volume of 50 ml) and liquid B (0.1 M sodium citrate solution, 1.47 g constant volume of 50 ml) at 4°C, avoiding light preservation, to 10 mg/ml solution configured to STZ before use. Rabbit antiphosphorylation signal transducers and activators of transcription-3 (STAT3) antibodies (batch number: GR287047-7) were purchased from Abcam Co. (Cambridge, United Kingdom).

### 2.2. Establishment of Mouse Models

**Figure 1:**
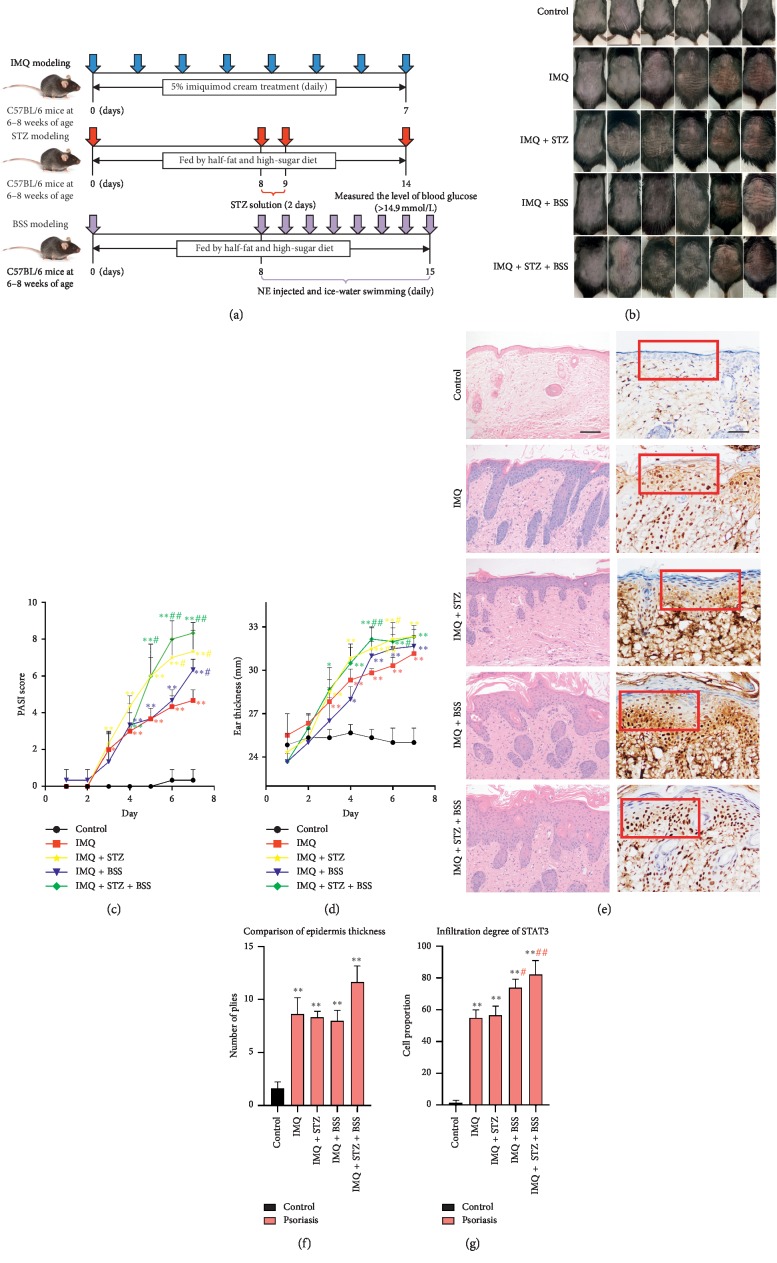
Induction methods and establishment of mouse psoriasis models. (a) Induction methods involving IMQ, STZ, and BSS. (b) After induction, skin lesions on the back of mice in each group were recorded for seven days. (c) After induction, the PASI score of back-skin lesions in each group was determined for seven days. (d) After induction, ear thickness in each group was measured for seven days. (e) Left: pathological sections of mouse back skin (H&E staining; magnification, ×100; scale bar, 100 *μ*M) showed thickening of the epidermis in each group on the last day of induction. Right: immunohistochemical analysis of mouse back skin (magnification, ×200; scale bar, 50 *μ*M); brown-yellow granule cells indicate STAT3-positive cells. (f) Comparison of epidermis thickness on the last day of induction. (g) Infiltration degree of STAT3 on the last day of induction. Compared with the control group, ^*∗∗*^*p* < 0.01, ^*∗*^*p* < 0.05; compared with the IMQ group, ^##^*p* < 0.01, ^#^*p* < 0.05.

The model establishment methods are shown in Figure 1(a).

#### 2.2.1. IMQ Group

The back hair of the mice was shaved, and IMQ cream was applied to the skin on their ears and back (1 cm^2^ of skin; 0.01 g IMQ cream was applied evenly using a rubber hose) once a day for seven days [13]. Photographs were taken daily to record changes in the skin lesion on the back, and the thickness of the ears was measured.

#### 2.2.2. IMQ + STZ Group

Mice were fed a half-fat and high-sugar diet (84.5% basic feed with 5% yolk powder, 0.5% cholesterol, 5% lard, and 5% sucrose) for one week. STZ solution was administered via intraperitoneal injection to mice at a dose of 100 mg/kg once daily for two days, and blood glucose level was measured on the fifth day. An obvious increase in blood glucose level indicated successful model establishment. Otherwise, the dose was administered again on the fifth day until blood glucose level reached >14.9 mmol/l. The day after blood glucose level reached >14.9 mmol/l, and IMQ cream was applied to the skin of the ears and back of the mice, with the same method as for the IMQ group. Starting from the first day of IMQ cream application, the skin lesions were photographed and recorded daily, ear thickness was measured, and body weights were recorded.

#### 2.2.3. IMQ + BSS Group

Mice were fed a half-fat and high-sugar diet (84.5% basic feed with 5% yolk powder, 0.5% cholesterol, 5% lard, and 5% sucrose) for one week. NE was administered via intraperitoneal injection in mice at a dose of 0.1 ml/100 g (0.1 g/l NE was prepared in normal saline). After 15 to 20 min, the mice were induced to swim in ice water at 4°C for 5 min once a day for 8 days. On the second day of ice-water swimming, IMQ cream was applied to the skin of the ears and back of the mice, using the same method as for the IMQ group. Starting from the first day of IMQ cream application, skin lesions were photographed and recorded daily, ear thickness was measured, body weights were recorded, and changes in mouse hair color, nails, tongue, and activity were observed every day.

#### 2.2.4. IMQ + STZ + BSS Group

The psoriasis induction method was the same as for the IMQ group, and the induction methods of blood stasis syndrome and metabolic disorder were the same as for the IMQ + BSS and IMQ + STZ groups, respectively. During the eight consecutive days of model induction, skin lesions were photographed and recorded daily, ear thickness was measured, and body weights were recorded.

#### 2.2.5. Specimen Collection

The experimental cycle was approximately 15 days. The mice were fasted but allowed water for 12 h before specimen collection. At 8 a.m. on the next day, 2 ml blood was extracted via the eyeball: 1 ml was injected into an anticoagulant test tube, and the other 1 ml was injected into a coagulant test tube. Blood samples were allowed to stand for 4°C for 1 h and then centrifuged in a refrigerated centrifuge at 4°C and 1800 rpm/min for 5 min. The supernatant was then collected and stored in a refrigerator at −80°C until analysis. The mice were sacrificed in a euthanasia box with the flow rate of CO_2_ into the box maintained at 20% volume of the box per minute. Specimens of the bilateral ears, back skin, and aortic arch were collected using surgical scissors and fixed in 100 ml/l paraformaldehyde solution. Paraffin blocks of specimens were then prepared routinely for analysis.

#### 2.2.6. Statistical Method

The data were analyzed by using the SPSS.21.0 statistical software (International Business Machines Corporation, Armonk, NY, USA). The data were expressed as (*x̄* ± *s*). Values of *p* < 0.05 indicated a statistical difference, and *p* < 0.01 indicated a significant difference. A group sample *t*-test was used for comparison between groups.

## 3. Results

### 3.1. Characteristics of Back-Skin Lesions and Ear Thickness in the Animal Models

The pathological features of the back and changes in ear thickness at days 2 to 7 were observed in the mice. The IMQ group showed significant thickening of the back skin on the fourth day of induction; on the seventh day of induction, there were obvious scales and erythema on the back. In the IMQ + STZ group, the skin thickened on the second day of induction, and the back skin became hard after 6 and 7 days of induction, accompanied by patches, peeling, and descaling. This suggested that glucose and lipid metabolism imbalance may accelerate the development of psoriatic lesions. The IMQ + BSS group showed significant psoriatic skin lesions on the fourth day of induction. On the seventh day of induction, the mice showed clinical manifestations of blood stasis: pale and cold back skin, bluish purple tongue, dim eyeball color, petechiae in the tail, and a significant scaly and erythema lesion area on the back skin. The IMQ + STZ + BSS group showed thickening of the back skin on the third day of induction; at the end of induction, the skin lesions on the back were pale, peeling, and desquamated. The mice also showed blood stasis syndrome, which was slightly less severe than that in the IMQ + BSS group. Owing to the slow induction process of blood stasis syndrome, skin lesions in the early stage were not significant. The fastest psoriasis induction process was observed in the IMQ + STZ group, and the most prominent psoriatic skin lesions were observed in the IMQ + STZ + BSS group (Figure 1(b)).

The PASI score showed similar results (Figure 1(c)). The PASI score of the back-skin lesions in each group showed varying degrees of changes on the third day of induction. The PASI score of the IMQ + STZ group showed the fastest increase, whereas that of the IMQ + STZ + BSS group was the highest on the seventh day of induction, suggesting that psoriasis complicated with glucose and lipid metabolism disorders may aggravate the primary skin lesions of psoriasis. In addition, the PASI score of the IMQ + BSS group increased slightly faster than that of the IMQ group, suggesting that blood stasis syndrome may accelerate the manifestation of primary skin lesions in psoriasis (*p* < 0.01).

IMQ cream was applied to the mouse ears, and mouse ear thickness was measured on the first to seventh day (Figure 1(d)). We found that mouse ears in the IMQ, IMQ + STZ, IMQ + BSS, and IMQ + STZ + BSS groups were thicker than those in the Control group (*p* < 0.01). Thickening of the mouse ears was the fastest in the IMQ + STZ + BSS group, followed by the IMQ + STZ group. The ears of the IMQ + BSS group were slightly thicker than those of the IMQ group, suggesting the same conclusion as that suggested by the PASI score.

### 3.2. Histopathological Features of Back-Skin Lesions

Mouse back skin collected on day 7 was stained with H&E and observed at 100× magnification (Figure 1(e), left). The IMQ, IMQ + STZ, IMQ + BSS, and IMQ + STZ + BSS groups showed psoriatic pathological changes compared with the Control group (*p* < 0.01; Figure 1(f)), such as epidermal hyperkeratosis or hyperkeratosis, epidermal cluster-like hyperplasia, dermal papillary extension, dermal papillary capillary dilation, congestion, and inflammatory cell infiltration around the upper dermal vessels. The back-skin lesions of the Control group showed epidermal and spinous layers arranged in a regular order, and the spinous layer was composed of 3–5 layers of polygonal cells, with clear boundaries of the true epidermis, no inflammatory infiltration of lymphocytes, and no telangiectasias or congestion.

Using immunohistochemical staining and microscopy at 200x magnification (Figure 1(e), right), we observed cells with brown-yellow granules in the cytoplasm and nucleus, which were STAT3-positive cells. There were almost no STAT3-positive cells in the dermis of the Control group. However, compared with the Control group, the IMQ, IMQ + STZ, IMQ + BSS, and IMQ + STZ + BSS groups showed significantly increased STAT3 expression (*p* < 0.01; Figure 1(g)). We examined the histogram at ×200 magnification and calculated the score of STAT3-positive cells in each group: light yellow, 1 point; yellow and dark yellow, 2 points; and brown-yellow, 3 points. The score of STAT3-positive cells was the highest in the IMQ + STZ + BSS group; STAT3-positive cells in the IMQ + STZ + BSS and IMQ + BSS groups were darker than those in the IMQ group (*p* < 0.05), suggesting that glucose and lipid metabolism disorders and blood stasis syndrome promoted the proliferation and differentiation of keratinocytes (KCs), and that the expression of STAT3 was positively correlated with PASI score.

### 3.3. Indexes of Glycolipid Metabolism in Animal Models

#### 3.3.1. Changes in Serum Insulin, C-Peptide, and Blood Glucose Levels

**Figure 2:**
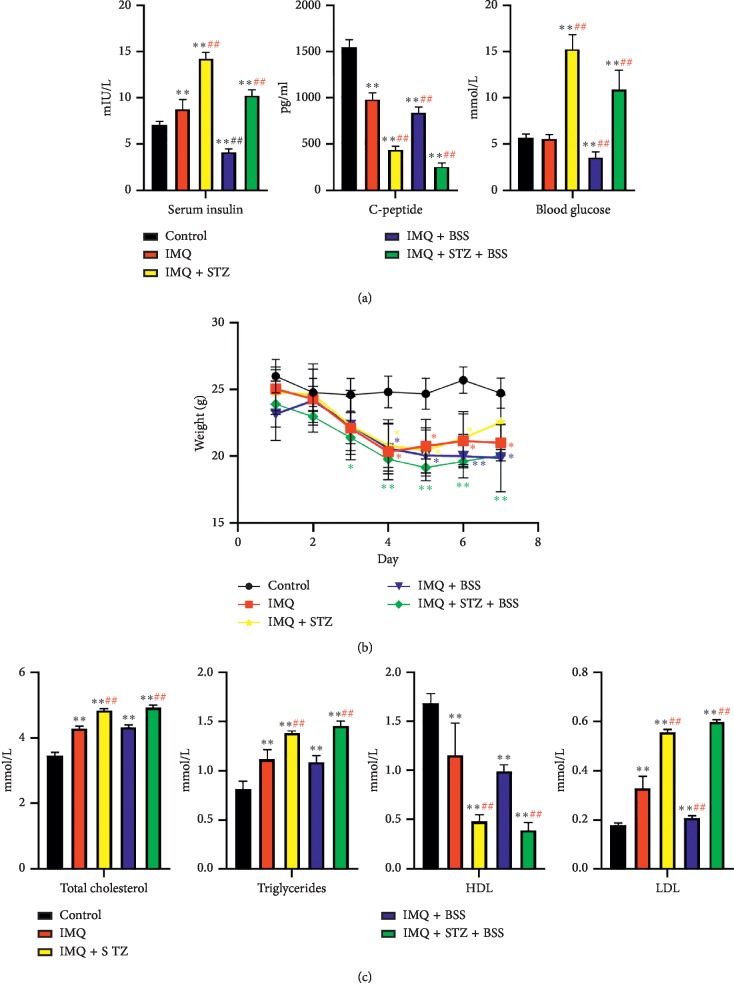
Levels of indicators of glucose and lipid metabolism. (a) Serum insulin, C-peptide, and blood glucose levels of each group on the last day of induction. (b) Body weights of each group measured for 7 days of IMQ induction. (c) Total cholesterol, triglyceride, HDL, and LDL levels of each group on the last day of induction. Compared with the control group, ^*∗∗*^*p* < 0.01, ^*∗*^*p* < 0.05; compared with the IMQ group, ^##^*p* < 0.01, ^#^*p* < 0.05.

Compared with the Control group at the end of induction, serum insulin levels significantly increased in the IMQ, IMQ + STZ, and IMQ + STZ + BSS groups (*p* < 0.01), but significantly decreased in the IMQ + BSS group (*p* < 0.01). Compared with those in the Control group, serum C-peptide levels significantly decreased in the IMQ + STZ, IMQ + BSS, and IMQ + STZ + BSS groups (*p* < 0.01), suggesting that islet dysfunction occurred in varying degrees in each model group after induction. Compared with those of the IMQ group, blood glucose levels significantly increased in the IMQ + STZ and IMQ + STZ + BSS groups (*p* < 0.01), but significantly decreased in the IMQ + BSS group (*p* < 0.01), suggesting that STZ induction increased blood glucose level. Moreover, blood glucose level in the IMQ + BSS group might have been too low owing to insufficient insulin secretion (Figure 2(a)).

#### 3.3.2. Changes in Weight, Total Cholesterol, Triglyceride, HDL, and LDL Levels

On the seventh day of induction, body weights of animals significantly decreased in the IMQ (*p* < 0.05), IMQ + BSS (*p* < 0.05), and IMQ + STZ + BSS (*p* < 0.01) groups, and insignificantly decreased in the IMQ + STZ group. Mouse body weights in the IMQ, IMQ + STZ, IMQ + BSS, and IMQ + STZ + BSS groups slightly increased at the fifth and sixth days of induction, which may be related to the induction treatments. At the end of the induction period, weight loss was the most obvious in the IMQ + BSS group, followed by the IMQ + STZ + BSS and IMQ + STZ groups, and the least obvious in the IMQ group (Figure 2(b)).

Compared with those in the Control group at the end of induction, the levels of total cholesterol, triglycerides, and LDL significantly increased in each model group (*p* < 0.01), whereas the levels of HDL were significantly decreased in each model group (*p* < 0.01), indicating that lipid metabolism disorders were prevalent in each model group (Figure 2(c)). However, the levels of total cholesterol, triglycerides, and LDL in the IMQ + STZ and IMQ + STZ + BSS groups were significantly higher than those in the IMQ group (*p* < 0.01), accompanied by decreased HDL levels (*p* < 0.01). This result suggested that compared with the Control group, the IMQ group had a certain degree of lipid metabolism disorder, and the model groups induced with STZ showed more severe lipid metabolism disorder than the IMQ group.

#### 3.3.3. Changes in Whole Blood Viscosity

**Figure 3:**
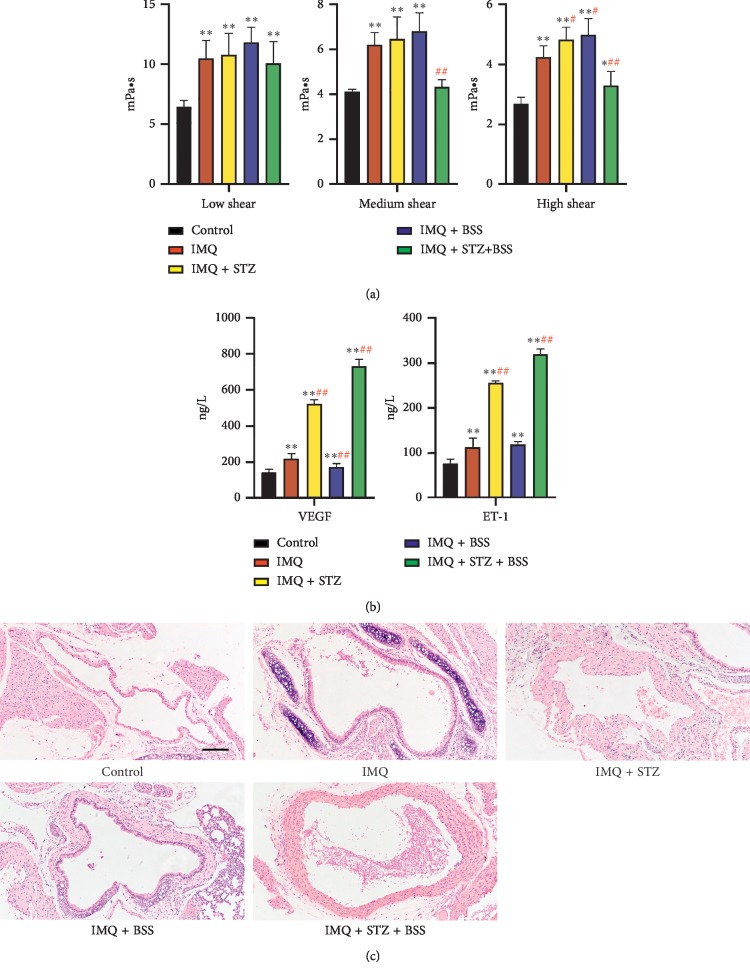
Levels of cardiovascular indicators. (a) Whole blood viscosity levels of each group on the last day of induction. (b) VEGF and ET-1 levels of each group on the last day of induction. (c) Pathological sections of mouse aortic arch from each group (H&E staining; magnification, ×100; scale bar, 100 *μ*M). Compared with the control group, ^*∗∗*^*p* < 0.01, ^*∗*^*p* < 0.05; compared with the IMQ group, ^##^*p* < 0.01, ^#^*p* < 0.05.

Compared with that in the Control group, the whole blood viscosity index of each model group was significantly increased, suggesting that psoriasis induction can lead to an increase in whole blood viscosity. Compared with that in the IMQ group, the whole blood viscosity of the IMQ + STZ + BSS group significantly decreased in both medium and high shear (*p* < 0.01). The high-shear whole blood viscosity level significantly increased in the IMQ + STZ and IMQ + BSS groups (*p* < 0.05), indicating that blood stasis syndrome as well as glucose and lipid metabolism disorders helped increase blood viscosity index (Figure 3(a)).

#### 3.3.4. Changes in VEGF and ET-1 Levels

Compared with those in the control group, VEGF and ET-1 levels were significantly increased in each model group at the end of induction (*p* < 0.01), especially in the IMQ + STZ and IMQ + STZ + BSS groups, suggesting that the three interventions (psoriasis, blood stasis syndrome, and glucose and lipid metabolism disorders) caused different degrees of microcirculation disorders. In addition, compared with that in the IMQ group, VEGF level in the IMQ + STZ, IMQ + BSS, and IMQ + STZ + BSS groups increased significantly (*p* < 0.01). The levels of ET-1 in the IMQ + STZ and IMQ + STZ + BSS groups significantly increased, further suggesting that glucose and lipid metabolism disorders and blood stasis syndrome aggravated psoriasis (Figure 3(b)).

#### 3.3.5. Changes in Dissection of Aortic Arch

On the seventh day of induction, the aortic arch was dissected from the mice and stained with H&E (Figure 3(c)). Microcopy analysis at ×100 magnification showed that the aortic arch wall markedly thickened in the IMQ + STZ, IMQ + BSS, and IMQ + STZ + BSS groups, and the intima in the IMQ + BSS group had a significantly granular bright red lining of visible lipid deposition and scattered calcification points. The aortic arch intima of the IMQ + STZ and IMQ + STZ + BSS groups showed accumulated lipid droplets, whereas that of the IMQ group showed neither lipid deposition nor lipid droplet accumulation. In addition, the aortic vessel lumen in the IMQ + STZ, IMQ + BSS, and IMQ + STZ + BSS groups was narrower and smaller than that in the Control group, with connective tissue hyperplasia on the outside of the wall.

## 4. Discussion

The idea of combining diseases and syndromes of CM has been used to study psoriasis animal model induction. At present, there are two published Chinese studies on the induction of blood stasis in psoriasis [14, 15], both of which used the ice-water swimming method to simulate blood stasis syndrome in combination with the characteristics of psoriasis skin lesions. The induction method is relatively mature, which induced the psoriasis-like lesions, and the blood stasis syndrome score was significantly higher than control. However, induction methods of specific syndromes of psoriasis complicated with other diseases (disorder of glucose and lipid metabolism) have not been reported.

Modern clinical research has demonstrated the existence of glucose and lipid metabolism disorders in patients with psoriasis, which is more likely to be complicated with fatty liver and diabetes [16, 17]. In psoriatic patients, lipid metabolism disorder is prone to cause metabolic syndrome [18], which is an aggregation of multiple clinical chronic disease risk factors, and its main clinical outcomes are cardiovascular and cerebrovascular diseases and diabetes. A prospective cohort study [19] showed that metabolic syndrome has a direct impact on the total mortality of the population and mortality due to cardiovascular disease, which can be used as an indicator to predict the total mortality of the population. Therefore, the significance of studying psoriasis with metabolic disorders cannot be ignored, and animal experiments are indispensable. A previous study [20] confirmed the existence of metabolic disorders in IMQ-induced psoriasis animal models; hyperglycemia has been shown to be closely associated with psoriasis, mainly through the expression of IL-17. In this study, ice-water swimming was used to simulate blood stasis syndrome, and STZ was used to induce type II diabetes; this was the first attempt to explore psoriasis with blood stasis syndrome complicated with glucose and lipid metabolism disorder.

Our results showed that, compared with the Control group, thickening of mouse dorsal skin, increased erythema and scaling, and increased PASI score were observed in the groups treated with IMQ cream, indicating successful establishment of psoriasis models. Half-fat diet was used to induce obesity and insulin resistance in mice [21], and intraperitoneal injection of a chain urea toxin (STZ) was applied to destruct pancreatic islet *β* cells in the mice [22], thus generating a condition similar to human type II diabetes. In the established mouse models, laboratory indicators, including C-peptide, glucose, total cholesterol, triglyceride, and LDL levels, increased, whereas HDL levels decreased, indicating the successful establishment of mouse models of psoriasis with glucolipid metabolic disorder. Notably, serum insulin levels were high in groups treated with STZ (Figure 2(a)). STZ-induced diabetic modeling is an accepted method in which pancreatic islet *β* cells are destroyed, leading to islet dysfunction and insufficient insulin secretion. In our study, insulin levels in the non-STZ groups were slightly lower than those in the STZ groups, and blood glucose levels of STZ mice were elevated significantly, which led to insulin resistance and induce type II diabetes. These results may indicate that our IMQ-STZ models were undergoing a transition from an insulin-resistant state to induce type II diabetes [23]. The blood stasis group received daily injection of NE to induce chronic endothelial cell injury and establish chronic blood stasis models; moreover, ice-water swimming resulted in Yang deficiency and warm blood, which led to blood stasis. The laboratory indexes after induction showed that whole blood viscosity increased, suggesting the successful establishment of blood stasis models [24].

From the microscopic perspective, increased VEGF and ET-1 indexes verified that the internal environment of the IMQ, IMQ + STZ, IMQ + BSS, and IMQ + STZ + BSS groups all presented different degrees of microcirculation disorders. The pathological sections of the dorsal skin showed thickening of the spinous layer, and inflammatory infiltration was most significant in the IMQ + STZ + BSS group, whereas the IMQ + STZ and IMQ + BSS groups showed the same trend of thickening and infiltration, suggesting that both blood stasis syndrome and glucose and lipid metabolism disorders aggravated the skin lesions of psoriasis. Pathological sections showed that the intima of the aortic arch in the IMQ + STZ and IMQ + STZ + BSS groups presented lipid droplet aggregation, accompanied by narrowing of the vascular lumen and hyperplasia of connective tissue on the outside of the arch wall, whereas the intima of the aortic arch in the IMQ + BSS group showed lipid deposition and scattered calcification points, suggesting that both blood stasis syndrome and glucose and lipid metabolism disorders induced aortic blood flow obstruction.

STAT3, a specific index of psoriatic KC proliferation [25], is mainly expressed in epidermis layers (the base layer, stratum spinosum, granular layer, and transparent layer) other than in the corneous layer. It has been shown that the mRNA expression of STAT3 in psoriasis patients with BSS was significantly higher than that in healthy people [26]. Immunohistochemical analysis results in the IMQ + STZ + BSS group showed a significantly increased number of STAT3-positive cells; the IMQ, IMQ + STZ, and IMQ + BSS groups also showed an increased number of STAT3-positive cells, to different extents. Therefore, it was suggested that IMQ cream treatment led to abnormal proliferation of KCs, and that glucose and lipid metabolism disorder and blood stasis syndrome aggravated psoriasis symptoms.

From the perspective of CM, blood stasis is a key to the pathogenesis of psoriasis [10, 27]. Blood stasis syndrome is also a common syndrome of heart disease, diabetes, vertigo, and other diseases in CM; therefore, studying blood stasis syndrome complicated with glucose and lipid metabolism disorders is of great significance. Previous studies by our research group have found that psoriasis is related to the occurrence of hyperuricemia and chronic obstructive pulmonary disease [28, 29], and high-intensity exercise reduces the prevalence of psoriasis [30]. In this study, we compared models of psoriasis with blood stasis syndrome, psoriasis complicated with glucose and lipid metabolism disorders, and psoriasis complicated with both blood stasis syndrome and glucolipid metabolic disorder in terms of aortic tissue pathology, hemorheology, VEGF and ET-1 levels, and other indexes. Our results were close to those observed in clinical practice, as expected, and close to those in human diseases. Thus, our models could be used to explore the pathogenesis of psoriasis and serve as an experimental basis of pharmacodynamics research.

## 5. Conclusions

We established mouse models of psoriasis with blood stasis syndrome complicated with glucose and lipid metabolism disorders, which presented the clinical characteristics specified in CM. Thus, our models could be used in studies investigating psoriasis complicated with glucose and lipid metabolism disorders.

## Data Availability

All data generated during this study are included in this published article and its supplementary information files.
